# Late entry into HIV care: lessons from Brazil, 2003 to 2006

**DOI:** 10.1186/1471-2334-12-99

**Published:** 2012-04-24

**Authors:** Alexandre Grangeiro, Maria Mercedes Loureiro Escuder, Julio Cesar Rodrigues Pereira

**Affiliations:** 1 Departamento de Medicina Preventiva da Faculdade de Medicina da Universidade de São Paulo, São Paulo, Brazil; 2 Instituto de Saúde. Secretaria de Estado da Saúde de São Paulo, São Paulo, Brazil; 3 Departamento de Epidemiologia da Faculdade de Saúde Pública da Universidade de São Paulo, São Paulo, Brazil

**Keywords:** AIDS, Health policy, Late HIV diagnosis, Risk, Brazil

## Abstract

**Background:**

To ascertain the population rates and proportion of late entry into HIV care, as well as to determine whether such late entry correlates with individual and contextual factors.

**Methods:**

Data for the 2003–2006 period in Brazil were obtained from public health records. A case of late entry into HIV care was defined as one in which HIV infection was diagnosed at death, one in which HIV infection was diagnosed after the condition of the patient had already been aggravated by AIDS-related diseases, or one in which the CD4^+^ T-cell count was ≤ 200 cells/mm^3^ at the time of diagnosis. We also considered extended and stricter sets of criteria (in which the final criterion was ≤ 350 cells/mm^3^ and ≤ 100 cells/mm^3^, respectively). The estimated risk ratio was used in assessing the effects of correlates, and the population rates (per 100,000 population) were calculated on an annual basis.

**Results:**

Records of 115,369 HIV-infected adults were retrieved, and 43.6% (50,358) met the standard criteria for late entry into care. Diagnosis at death accounted for 29% (14,457) of these cases. Late entry into HIV care (standard criterion) was associated with certain individual factors (sex, age, and transmission category) and contextual factors (region with less economic development/increasing incidence of AIDS, lower local HIV testing rate, and smaller municipal population). Use of the extended criteria increased the proportion of late entry by 34% but did not substantially alter the correlations analyzed. The overall population rate of late entry was 9.9/100,000 population, specific rates being highest for individuals in the 30–59 year age bracket, for men, and for individuals living in regions with greater economic development/higher HIV testing rates, collectively accounting for more than half of the cases observed.

**Conclusions:**

Although the high proportion of late entry might contribute to spreading the AIDS epidemic in less developed regions, most cases occurred in large cities, with broader availability of HIV testing, and in economically developed regions.

## Background

The effectiveness of AIDS treatment programs is severely affected by the proportion of HIV-infected individuals who do not enter into clinical care in a timely manner (i.e., who enter late). This is because HIV-infected individuals who are under treatment are less likely to transmit the virus to others, not only because such individuals tend to adopt safe sex practices but also because the use of antiretroviral therapy (ART) can reduce the risk of sexual transmission of HIV by more than 90% [1,2]. Late entry into care is also strongly associated with premature death among HIV-infected individuals [3-7], accounting for approximately 40% of all AIDS-related deaths [8]. Late entry into HIV care also has negative consequences for health care systems, increasing costs due to the medications and highly complex procedures required in order to treat individuals who are in the advanced stages of HIV infection when they enter treatment [9,10]. To minimize this problem, there have been numerous initiatives aimed at promoting early diagnosis and the initiation of clinical treatment in the early stages of infection [11-15], particularly through encouraging voluntary testing [11,13,14] and by strengthening the cooperation between and among centers for the diagnosis and treatment of HIV-infected individuals [13,14]. Nevertheless, the proportion of individuals who have sought treatment late remains high—over 30% in high income countries [16-23] and over 70% in African countries [9,24]. The main hindrances to the success of these initiatives are related to the individual [12,13,25,26], the social context [25-27], and the way in which the health care services are organized [19,28-30].

Although differing in their methodological approaches [31], studies of late entry into HIV care have provided, for different contexts, a broad range of estimates of the proportion of such late entry and have identified different associated risk factors [16,19-23,32]. Therefore, there is a need for further knowledge about the absolute risk of late entry, both at the population level and in social segments exposed to certain risk factors (such as advanced age, male gender, and a low level of local economic development). In low- and middle-income countries, the proportion of individuals entering HIV care late has often been evaluated on the basis of the time at which ART is initiated, rather than the actual time of entry into care [24,33,34]. This strategy has two drawbacks. First, it ignores the fact that other therapeutic and prophylactic interventions should be undertaken prior to the initiation of ART. Second, the criterion for starting ART has changed over time, and there is a trend toward earlier prescription of ART. Therefore, when late entry into care is defined by the initiation of ART, it is difficult to make comparisons over time and across studies.

In a recent study [8], we evaluated data related to the overall population of HIV-infected individuals who entered care in Brazil between 2003 and 2006, estimating the proportion of individuals entering late into care and the impact that this has on the rates of AIDS mortality. In the present study, we sought to analyze the demographic and epidemiological characteristics related to late entry, estimating the absolute and relative risk, which we calculated for that population as a whole and for different social segments, using various clinical and immunological parameters. We have also analyzed the proportional contribution of different social segments to the total number of individuals entering late into HIV care in Brazil.

## Methods

To analyze the general population of HIV-infected individuals (over 15 years of age) who entered into care at public health care facilities in Brazil between 2003 and 2006, we obtained data from the information systems of the Brazilian National Ministry of Health. Specifically, we obtained data from the following: the *Sistema de Controle de Exames Laboratoriais da Rede Nacional de Contagem de Linfócitos CD4*^*+*^*/CD8*^*+*^*e Carga Viral* (SISCEL, Brazilian National CD4^+^/CD8^+^ T Lymphocyte Count and Viral Load Network Laboratory Test Control System) database—for the initial CD4^+^ T-cell counts (the national guidelines in Brazil state that an initial CD4^+^ T-cell count must be obtained at time of entry into HIV care); the *Sistema de Informação de Agravos de Notificação* (SINAN, Disease Notification Information System)—for information on the occurrence of AIDS-related diseases; the *Sistema de Informação sobre Mortalidade* (SIM, Mortality Information System)—for information on AIDS-related deaths (from death certificates); and the *Departamento de Informática do Sistema Único de Saúde* (DATASUS, Unified Health Care System Department of Information Technology)—for electronic records of treatment provided via the public health care system and contextual information on municipalities. The coverage of these systems ranges from 62.3% (SINAN) to 83% (SIM/SISCEL), as described in our earlier study [8], in which we also addressed the completeness of the data contained in the various databases.

To determine whether the databases employed included information on the maximum number of HIV-infected individuals entering into clinical treatment during the period under study, the SISCEL, SINAN, and SIM data were cross-referenced, by probabilistic record linkage, and the duplicate entries generated thereby were excluded [35]. The process of including cases in the study was described with a specific algorithm [8]. Cases in which data related to entry into care or mortality were incongruent were excluded from the analysis, as were those in which the initial CD4^+^ T-cell count was delayed by more than six months, which would have misrepresented the initial immunological status.

A case of late entry into HIV care was typically defined as one in which HIV infection was diagnosed at death, one in which HIV infection was diagnosed after the condition of the patient had already been aggravated by AIDS-related diseases, or one in which the CD4^+^ T-cell count was ≤ 200 cells/mm^3^ at the time of diagnosis. Those were the standard criteria. In addition, we considered an extended set of criteria, in which the first two criteria were the same but the initial CD4^+^ T-cell count was ≤ 350 cells/mm^3^, as well as a stricter set, in which the first two criteria were the same but the initial CD4^+^ T-cell count was ≤ 100 cells/mm^3^. A case of HIV diagnosed at death was defined as a case in which the diagnosis was made within 20 days before death. The date of entry into care was defined as that of the earliest record found in any of the data sources. Data related to sex, age, and place of residence were collated into an analysis database, with the following hierarchy: SINAN, SIM, then SISCEL. The category of HIV transmission was obtained exclusively for AIDS cases reported to SINAN, because that information is not available in any of the other databases. We also analyzed contextual information (geopolitical region, HIV testing rate, and municipal population size), which were obtained from DATASUS.

Data were stratified by geopolitical region, because there are major differences among those regions in terms of human development, health care access, and trends in the AIDS epidemic. It has been shown that, in the northern and northeastern regions of Brazil, in comparison with the other regions of the country, there is a tendency toward an increase in the incidence of AIDS, the lowest per capita rates of medical visits, and the lowest per capita gross domestic product (GDP) [36].

The rate of HIV testing was assessed at the municipal level as the number of enzyme-linked immunosorbent assay and rapid tests performed at public health care facilities per thousand population in 2008. Based on these data, the HIV testing rate was categorized as follows: low (≤ 1 HIV test/1,000 population); medium (between 1.01 and 10 HIV tests/1,000 population); or high (> 10 HIV tests/1,000 population). The municipalities were categorized by population: < 100,000 inhabitants; 100,000-499,999 inhabitants; or ≥ 500,000 inhabitants.

Five categories of HIV transmission were considered: through exclusively female-to-male sexual contact (in heterosexual men); through exclusively male-to-female sexual contact (in heterosexual women); through male-to-male or male-to-female sexual contact (in homosexual/bisexual men); through injection drug use; and through transfusion of blood or blood products. Individuals fitting more than one category of transmission were classified using the following hierarchy: recipients of blood transfusions; injection drug users; homosexual/bisexual males; heterosexual individuals (males or females); and users of blood products.

The study protocol was approved by the Research Ethics Committee of the São Paulo State STD and AIDS Referral and Training Center, located in the city of São Paulo, Brazil. Because patient data were collected from the Brazilian Ministry of Health (NMH) national information systems and analyzed anonymously after the systems had been cross-referenced, informed consent was not necessary.

### Data analysis

The proportion and population rates of late entry into HIV care were estimated for the standard, extended, and strict definitions. The distribution of those rates was examined by sex, age bracket, geopolitical region, and HIV testing rate. Population rates (per 100,000 population) were calculated on an annual basis, the denominator being the adult population at risk of late entry into HIV care. Individuals already under care at the beginning of a given year, and those in whom HIV had been diagnosed in a timely manner during that year, were not included in the annual denominator. The averages for the study period were used in the analyses.

Poisson regression with a log-link function was used in order to assess effect as risk ratios (RR) and the respective 95% confidence intervals (95% CIs), using the standard late entry criteria as the reference. Individual and contextual variables with a p < 0.05 were included in the model, whereas those with a p < 0.10 were excluded. The category of HIV transmission was analyzed with a separate Poisson regression model.

The impact of the changes in the scenario resulting from the application of the three different sets of criteria (standard, strict and extended) was examined by determining the differences in magnitude of the proportions and in the profile (demographic and epidemiological characteristics) of the individuals included under each set of criteria.

## Results

We identified 115,369 HIV-infected individuals who entered into care in Brazil between 2003 and 2006. Of those individuals, 56.3% were male, 66.0% were between 30 and 59 years of age, and 50.7% resided in the southeastern region of the country (Table 1). The annual number of new cases ranged from 24,487 (in 2003) to 31,941 (in 2005), and the annual average number of new patients for the entire period was 28,842.

**Table 1:** Proportion of late entry into HIV care, as defined by the standard criterion, with adjusted risk ratios. Brazil (2003–2006)

**Characteristic**		**N**	**%**	**Proportion of late entry**	**Risk ratio**	**Adjusted risk ratio**	**95% confidence interval**		**p**
							**Min**	**Max**	
Brazil		115,369	100	43.6	-	-	-	-	-
Geopolitical region	South	22,229	19.3	40.8	1	1	-	-	
	North	6,270	5.4	53.4	1.31	1.33	1.29	1.37	< 0.001
	Northeast	17,448	15.1	48.1	1.18	1.19	1.16	1.21	< 0.001
	Central-West	7,997	6.9	47.1	1.15	1.16	1.13	1.20	< 0.001
	Southeast	58,499	50.7	43.1	1.06	1.05	1.03	1.07	< 0.001
	No data	2,926	2.5						
Size of municipality (number of inhabitants)	≥ 500,000	46,835	40.6	42.6	1	1	-	-	
	100,000-499,999	33,339	28.9	43.0	1.01*	1.04	1.02	1.06	< 0.001
	< 100,000	30,429	26.4	46.3	1.09	1.08	1.06	1.10	< 0.001
	No data	4,766	4.1						
HIV testing rate^†^	High	89,550	77.6	42.7	1	1	-	-	
	Medium	5,063	4.4	46.8	1.10	1.06	1.03	1.10	<0.001
	Low	15,990	13.9	48.5	1.14	1.09	1.06	1.11	< 0.001
	No data^‡^	4,766	4.1		1				
Sex	Female	50,354	43.6	35.7		1	-	-	
	Male	64,932	56.3	49.8	1.40	1.33	1.31	1.35	< 0.001
	No data	83	0.1						
Age bracket	15-29 years	36,201	31.4	30	1	1	-	-	
	30-59 years	76,134	66	49.5	1.65	1.60	1.58	1.63	< 0.001
	≥ 60 years	3,034	2.6	59.3	1.98	1.91	1.85	1.98	< 0.001
HIV transmission category^§^	Heterosexual women	15,665	41.9	-	1	1	-	-	
	Injection drug user	2,997	8.0	-	1.18	1.18	1.15	1.22	< 0.001
	Heterosexual men	11,467	30.7	-	1.21	1.17	1.15	1.19	< 0.001
	Blood transfusion	107	0.3	-	1.16	1.15	1.01	1.32	0.039
	Homosexual/bisexual men	7,155	19.1	-	1.06	1.04	1.02	1.07	< 0.001

*Not significant; ^†^High = > 10 tests/1,000 population, Medium = from 1.01 to 10 tests/1,000 population, Low = ≤ 1 test/1,000 population; ^‡^No information available on city of residence; ^§^The categories of transmission were analyzed with a separate Poisson regression model, including the 37,391 individuals for whom information on transmission category was available. Control of confounding variables = region, HIV testing rate, and age. Population size was disregarded as a misleading variable as it did not show statistical significance in this regression model.

We excluded 18,638 (13.9%) cases in which the initial CD4^+^ T-cell count was obtained six months or more after entry into care, as well as 42 (0.03%) cases in which the information related to the date of entry into care was inconsistent. The demographic characteristics of the excluded cases were similar to those of the cases included in the analysis [8].

### Proportion and relative risk of late entry into HIV care

As can be seen in Table 1, the overall proportion of late entry into HIV care, according to the standard criterion, was 43.6% (50,358 individuals) between 2003 and 2006. We detected a slight, but statistically significant, decrease between the first and last years of the period studied (from 43.5% to 41.6%, p < 0.001).

Table 1 also shows that the relative risk of late entry into HIV care was highest for the ≥ 60 year age bracket (RR: 1.91, 95% CI: 1.85-1.98); for the northern and northeastern regions (RR: 1.33, 95% CI: 1.29-1.37 and RR: 1.19, 95% CI: 1.16-1.21, respectively); and for males (RR: 1.33, 95% CI: 1.31-1.35). At the municipal level, lesser, but statistically significant, effects were observed for low HIV testing rate (RR: 1.09, 95% CI: 1.06-1.11) and having ≤ 100,000 inhabitants (RR: 1.08, 95% CI: 1.06-1.10).

Information on the HIV transmission category was available for 37,391 cases during the period under study. Of those, 41.9% were in heterosexual women, 30.7% were in heterosexual men, 19.1% were in homosexual/bisexual men, 8.0% were in injection drug users, and 0.3% were in individuals who had received transfusions of blood or blood products (Table 1). The highest risk ratios were found for injection drug users (RR = 1.18, 95% CI: 1.15-1.22) and heterosexual men (RR = 1.17, 95% CI: 1.15-1.19). Marginal risk ratios were observed for being a homosexual/bisexual men (RR = 1.04; 95% CI: 1.02-1.07) and for having received a blood transfusion (RR = 1.15, 95% CI: 1.01-1.32).

As a corollary of the associations observed, the stratified analysis of the proportion of late entry into HIV care showed marked variation depending on the social segment analyzed (Table 2). The lowest proportion (about half of the national average) was among women in the 15–29 year age bracket residing in the southern or southeastern region of the country. Among such women, the proportions were 20.2% for those residing in the southern region and 22.2% for those residing in the southeastern region. For individuals (of either sex) in the ≥ 60 year age bracket and residing in the northern, northeastern, and central-west regions of the country, the proportion was above 60%.

**Table 2:** Stratified analysis of the proportion of late entry into HIV care, as defined by standard and extended criteria, and percentage increase with the use of the extended criterion. Brazil (2003–2006)

**Characteristic**	**Female by age bracket**				**Male by age bracket**				**Total by age bracket**			
	**15 - 29**	**30 - 59**	**≥ 60**	**Total**	**15 - 29**	**30 - 59**	**≥ 60**	**Total**	**15 - 29**	**30 - 59**	**≥ 60**	**Total**
**Standard criterion**												
Brazil	23.1	42.9	55.1	35.7	37.6	53.9	61.9	49.8	30.0	49.5	59.3	43.6
Geopolitical region												
North	33.8	54.9	65.9	45.7	50.5	63.0	60.9	58.8	42.4	60.0	62.5	53.4
Northeast	28.1	47.9	62.0	40.1	45.2	57.3	63.8	54.0	36.5	53.8	63.2	48.1
Central-West	24.3	49.1	60.5	38.8	37.2	59.4	69.2	53.3	30.5	55.5	66.0	47.1
Southeast	22.2	42.2	55.3	35.9	35.1	52.5	61.9	48.5	28.6	48.4	59.3	43.1
South	20.2	38.8	50.6	31.4	36.0	54.0	62.0	49.5	26.6	47.6	57.6	40.8
HIV testing rate^1^												
High	22.5	42.0	54.9	35.1	36.2	52.6	61.3	48.5	29.2	48.4	58.8	42.7
Medium	26.4	45.5	63.2	38.6	41.8	58.1	55.4	54.0	32.9	52.8	58.3	46.8
Low	25.3	47.3	56.2	38.7	45.5	61.3	65.5	57.2	34.1	55.4	62.4	48.5
**Extended criterion**												
Brazil	39.4	58.5	66.8	51.5	52.4	68.1	75.6	64.1	45.6	64.3	72.2	58.6
Geopolitical region												
North	55.7	74.7	81.8	66.4	67.6	78.9	76.1	75.1	61.8	77.4	77.9	71.5
Northeast	43.7	62.8	71.5	55.2	60.6	71.0	79.2	68.3	52.0	67.9	76.6	62.7
Central-West	43.0	65.0	73.7	55.9	53.7	74.2	82.7	68.6	48.2	70.8	79.4	63.2
Southeast	37.4	57.3	66.4	50.9	49.3	66.3	74.1	62.3	43.3	62.7	71.1	57.4
South	37.9	56.7	65.4	49.1	51.7	69.1	78.1	64.8	43.5	63.9	73.2	57.3
HIV testing rate^*^												
High	39.1	58.1	67.5	51.3	51.4	67.1	75.3	63.1	45.1	63.5	72.2	58.0
Medium	42.2	61.0	73.7	54.2	56.0	72.7	66.2	68.4	48.1	67.7	68.9	61.7
Low	41.8	61.9	66.4	53.9	60.5	74.4	80.0	70.8	49.9	69.1	75.5	62.9
**Percentage increase with extended criterion**												
Brazil	70.6	36.6	21.2	44.4	39.4	26.3	22.0	28.7	52.0	29.9	21.8	34.4
Geopolitical region												
North	65.1	36.1	24.1	45.3	33.9	25.2	25.0	27.7	45.8	28.8	24.6	33.9
Northeast	55.5	31.1	15.3	37.7	34.1	23.9	24.3	26.3	42.5	26.4	21.2	30.4
Central-West	77.4	32.4	21.8	44.1	44.4	25.1	19.5	28.7	58.0	27.6	20.3	34.2
Southeast	68.5	35.5	20.1	41.8	40.5	26.3	19.7	28.7	51.4	29.5	19.9	33.2
South	87.6	45.9	29.1	56.4	43.6	28.0	26.0	30.9	63.2	34.2	27.1	40.2

^*^High = > 10 tests/1,000 population; Medium = from 1.01 to 10 tests/1,000 population; Low = ≤ 1 test/1,000 population.

### Population rate of late entry into HIV care

The average population rate of late entry into HIV care observed for Brazil as a whole was 9.9 cases/100,000 population (Table 3). Regardless of sex and geopolitical region of residence, the average population rate was found to be highest among individuals in the 30–59 year age bracket (15.5 cases/100,000 population), in which it was 5.3 times higher than was that found for those in the ≥ 60 year age bracket (2.9/100,000 population).

**Table 3:** Population rates* of late entry into HIV care (100,000 population), as defined by standard and extended criteria. Brazil (2003–2006)

**Characteristics**	**Female by age bracket**				**Male by age bracket**				**Total by age bracket**			
	**15 to 29**	**30 to 59**	**≥ 60**	**Total**	**15 to 29**	**30 to 59**	**≥ 60**	**Total**	**15 to 29**	**30 to 59**	**≥ 60**	**Total**
**Standard criterion**												
Brazil	4.4	10.5	1.8	7.0	6.5	21.3	4.1	13.3	5.3	15.5	2.9	9.9
Geopolitical region												
North	4.5	9.9	1.8	6.6	7.1	18.5	3.5	11.8	5.8	14.3	2.7	9.2
Northeast	2.8	6.5	0.9	4.2	4.5	13.9	2.2	8.3	3.7	10.0	1.5	6.2
Central-West	4.6	10.6	2.7	7.3	6.6	22.1	5.6	13.9	5.6	16.3	4.1	10.6
Southeast	4.3	11.7	2.2	7.7	6.8	23.5	4.9	14.8	5.5	17.4	3.4	11.1
South	6.4	11.6	2.2	8.4	7.7	23.1	5.2	15.3	7.0	17.2	3.5	11.8
HIV testing rate^§^												
High	5.2	12.4	2.3	8.3	8.1	26.2	5.5	16.7	6.5	18.7	3.8	12.1
Medium	4.4	9.9	1.7	6.7	5.2	18.0	2.7	11.0	4.6	13.8	1.9	8.6
Low	2.3	5.3	0.7	3.5	3.1	9.6	1.7	5.9	2.6	7.4	1.2	4.6
**Extended criterion**												
Brazil	7.2	14.1	2.3	9.9	8.8	26.4	5.1	16.8	8.0	20.0	3.6	13.2
Geopolitical region												
North	7.4	13.5	2.3	9.6	9.4	23.2	4.4	15.0	8.4	18.5	3.4	12.3
Northeast	4.4	8.5	1.1	5.8	6.0	17.2	2.7	10.5	5.2	12.6	1.8	8.1
Central-West	8.1	14.0	3.3	10.5	9.5	27.7	6.7	17.9	8.8	20.8	5.0	14.2
Southeast	7.2	15.9	2.6	10.9	9.5	29.6	5.9	19.1	8.4	22.5	4.0	14.8
South	11.9	16.9	2.8	13.2	11.1	29.6	6.6	20.0	11.5	23.1	4.5	16.5
HIV testing rate^§^												
High	8.7	16.8	3.0	11.9	11.3	33.0	7.0	21.4	10.0	24.5	4.7	16.5
Medium	6.8	13.4	1.6	9.3	6.6	22.2	2.9	13.6	6.7	17.7	2.2	11.4
Low	3.7	7.0	0.9	4.8	4.0	11.4	2.2	7.1	3.8	9.2	1.5	6.0

*Average for 2003 to 2006.

^§^High = > 10 tests/1,000 population; Medium = from 1.01 to 10 tests/1,000 population; Low = ≤ 1 test/1,000 population.

The average population rates of late entry into HIV care tended to rise in parallel with regional increases in per capita GDP and in parallel with regional decreases in the incidence of AIDS. Consequently, the average rate was highest in the southern region (11.8/100,000 population) and lowest in the northeastern region (6.2/100,000 population.

**Figure 1:**
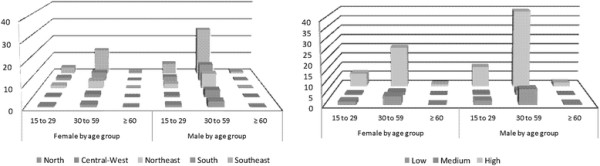
**Proportional distribution of the total numbers of cases of late entry into HIV care, as defined by the standard criterion.** Brazil (2003–2006).

The population rate of late entry into HIV care tended to decrease in parallel with that of HIV testing rates. This tendency was observed systematically in all of the social segments studied. In cities, where HIV testing rates are higher, the population rate of late entry into HIV care (12.1/100,000 population) was 22% higher than the national average. As a corollary of this population rate tendency, the largest proportions of individuals entering late into HIV care were in areas where the HIV testing rate was highest, were in the 30–59 year age bracket, were male, and resided in certain geopolitical regions (those with a higher GDP and a decreasing incidence of AIDS), accounting for 79.1%, 74.9%, 64.2%, and 50.6%, respectively, of all cases of late entry into HIV care between 2003 and 2006 (Figure 1).

### Stricter and extended criteria

When late entry into HIV care was defined according to the stricter criterion (Table 4), there were 34,088 cases (29.5% of all reported cases). Of those, 19,631 (17.0% of all reported cases) had met the criterion on the basis of the initial CD4^+^ T-cell count, and 14,457 (12.5% of all reported cases) were cases of diagnosis at death. Individuals in whom late entry was classified according to the stricter criterion accounted for 67.7% of those in whom it was classified according to the standard criterion. Among those in whom late entry was classified according to the stricter criterion, there was a predominance of men, individuals in the ≥ 60 year age bracket, and individuals residing in certain geopolitical regions (those with a lower GDP and an increasing incidence of AIDS).

**Table 4:** Proportion of late entry into HIV care, as defined by the stricter criterion. Brazil (2003–2006)

**Characteristics**	**Female by age bracket**				**Male by age bracket**				**Total by age bracket**			
	**15 - 29**	**30 - 59**	**≥ 60**	**Total**	**15 - 29**	**30 - 59**	**≥ 60**	**Total**	**15 - 29**	**30 - 59**	**≥ 60**	**Total**
Brazil	13.9	28.7	37.6	23.3	25.2	37.4	44.8	34.4	19.3	33.9	42.1	29.5
Geopolitical region												
North	20.0	35.5	38.6	28.6	34.8	44.2	44.6	41.1	27.6	40.9	42.6	35.9
Northeast	18.6	33.7	40.9	27.7	31.8	41.6	44.2	38.8	25.1	38.6	43.0	34.1
Central-West	14.0	32.5	40.8	24.8	24.2	40.3	47.4	35.8	18.9	37.4	45.0	31.2
Southeast	12.9	28.6	39.6	23.7	23.3	36.3	47.3	33.4	18.1	33.2	44.3	29.3
South	11.7	24.3	31.2	19.2	23.1	36.3	41.0	33.0	16.4	31.3	37.2	26.4

When late entry into HIV care was defined according to the extended criterion (Table 2), there were 67,615 cases (58.6% of all reported cases). The proportions were highest among residents of the geopolitical regions with a lower GDP and an increasing incidence of AIDS, especially among men in the 30–59 year age bracket, as well as among men and women in the ≥ 60 year age bracket. In those social segments, the proportions were approximately 80% when the extended criterion was applied.

In comparison with late entry classified according to the standard criterion, the use of the extended criterion resulted in the inclusion of an additional 17,257 individuals (a 34.4% increase). The largest proportional increase (of over 70%) was observed among the population segments with the lowest proportion of late entry into HIV care when the standard criterion was applied, among which there was a predominance of women in the 15–29 year age bracket, especially in the geopolitical regions with a higher GDP and a decreasing incidence of AIDS.

When the stricter, standard, and extended criteria were compared in terms of the relative risk, the only differences observed were in the magnitude of the risk ratio, which trended discretely higher for the stricter criterion and discretely lower for the extended criterion. In addition, the categories presenting a marginal association when the standard criterion was applied lost their significance when the extended criterion was applied. That was especially true for the southeastern region, for municipalities with 100,000-499,999 inhabitants, and for being a homosexual/bisexual man (Table 5).

**Table 5:** Proportion of late entry into HIV care, as defined by the extended and stricter criteria, with adjusted risk ratios. Brazil (2003–2006)

**Characteristic**		**Extended Criterion**				**Stricter Criterion**			
		**Adjusted risk ratio**	**95% Confidence interval**		**p**	**Adjusted risk ratio**	**95% Confidence interval**		**p**
			**Min**	**Max**			**Min**	**Max**	
Geopolitical Region	South	1	-	-	-	1	-	-	-
	North	1.26	1.23	1.28	< 0.001	1.41	1.35	1.47	< 0.001
	Northeast	1.10	1.08	1.11	< 0.001	1.30	1.26	1.34	< 0.001
	Central-West	1.11	1.08	1.13	< 0.001	1.20	1.14	1.24	< 0.001
	Southeast	1.00	0.98	1.01	0.498	1.11	1.08	1.13	< 0.001
Size of municipality (number of inhabitants)	≥ 500,000	1	-	-	-	1	-	-	
	100,000-499,999	1.01	1.00	1.02	0.194	1.07	1.05	1.09	< 0.001
	< 100,000	1.02	1.01	1.04	0.005	1.10	1.07	1.13	< 0.001
HIV testing rate^†^	High	1	-	-	-	1	-	-	-
	Medium	1.05	1.03	1.08	< 0.001	1.11	1.06	1.16	<0.001
	Low	1.07	1.05	1.09	< 0.001	1.11	1.08	1.15	< 0.001
Sex	Female	1	-	-	-	1	-	-	-
	Male	1.20	1.19	1.21	< 0.001	1.40	1.37	1.42	< 0.001
Age	15-29 years	1	-	-	-	1	-	-	-
	30-59 years	1.39	1.37	1.40	< 0.001	1.70	1.66	1.74	< 0.001
	≥ 60 years	1.56	1.52	1.60	< 0.001	2.10	2.00	2.20	< 0.001
HIV Category of transmission^§^	Heterosexual women	1	-	-	-	1	-	-	-
	Injection drug user	1.07	1.05	1.09	< 0.001	1.15	1.09	1.21	< 0.001
	Heterosexual man	1.08	1.07	1.09	< 0.001	1.15	1.12	1.19	< 0.001
	Blood transfusion	1.11	1.03	1.19	0.004	0.96	0.73	1.27	0.793
	Homosexual/bisexual male	1.01	0.99	1.02	< 0.203	1.00	0.96	1.04	< 0.001

^†^High = > 10 tests/1,000 population; Medium = from 1.01 to 10 tests/1,000 population; Low = ≤ 1 test/1,000 population. ^§^The categories of transmission were analyzed with a separate Poisson regression model, including the 37,391 individuals for whom information on transmission category was available. Control of confounding variables = region, HIV testing rate, and age. Population size was disregarded as a misleading variable as it did not show statistical significance in this regression model.

## Discussion

Because the Brazilian information systems provide consistent data on cases of HIV infection, it has been possible to increase our knowledge of aspects that are rarely discussed in the literature. A particular concern is the lack of information regarding late entry into HIV care in middle-income countries. The results of the present study show that, in Brazil, at least 2 out of every 5 HIV-infected individuals entered late into care between 2003 and 2006. Of those, approximately 70% presented with severe clinical and immunological impairment, of whom approximately 30% were diagnosed only at death. In addition, the application of the extended criterion (i.e., the inclusion of individuals with an initial CD4^+^ T-cell count ≤ 350 cells/mm^3^) increased the number of individuals entering late into HIV care by approximately one third. Therefore, approximately 6 out of every 10 HIV-infected individuals who entered into care in Brazil did so without having the opportunity to receive ART when it would have been most effective, resulting in less protection against new cases of infection in others and potentially higher nationwide rates of AIDS progression and mortality [1,2,8]. Such elevated rates could be responsible for the lack of advances in policies aimed at combating AIDS between 2000 and 2010, a period during which the absolute and relative numbers of AIDS cases increased [37].

Taking as our point of reference the proportion of late entry into HIV care when the standard criterion was applied, we found that Brazil is in an intermediate position in comparison with other countries. Lower proportions have been reported for high-income countries and regions, such as Northern France and Brussels (31.3% in the 1997–2007 period) [16]; New Zealand (32.0% in the 2005–2009 period) [23]; the United Kingdom and Ireland (33.4% in 2003) [19]; the United States (36.4% in 2005) [22]; Barcelona (38.5% in the 2001–2009 period) [21]; and Italy (39.2% in the 1997–2000 period) [18]. However, higher proportions have been reported for low- and middle-income countries. In a cohort study involving 36,000 patients in 12 countries [24], the collective proportions were 77% for the countries in Africa, 78% for the countries in Asia, and 51% for the countries in South America. Proportions higher than that observed in Brazil have also been reported for Thailand (55% for the 2003–2004 period) [32] and for Vietnam (58.3% for the 2004–2005 period) [38]. In Brazil, the reported proportion of late entry into HIV care has ranged from 42% in a nationwide study defining entry into care as the initiation of ART [34] to 68% in a study evaluating outpatients in the Brazilian state of Minas Gerais [33]. Comparisons across studies should be undertaken with caution, because different researchers adopt different definitions of late entry and evaluate different populations [31].

It is of note that we have combined a population-based risk assessment (based on population rates of late entry into HIV care) with a relative risk assessment (based on proportions and risk ratios for such late entry). Using this approach, we obtained results that differ, in many aspects, from those reported in the literature [16,19-23,32,38]. One such discrepancy is the fact that, when analyzing the population-based risk (assessed by late entry rate), we found that, for individuals in the 30–59 year age bracket residing in geopolitical regions with a higher GDP, a decreasing incidence of AIDS, and the highest HIV testing rates, the risk of late entry into HIV care was 35% higher than the national average, despite the lower relative risk and lower proportions of late entry found for that same group. Such individuals accounted for approximately 70% of the total number of cases of late entry in Brazil between 2003 and 2006. These results can be understood within the context of the fact that the population-based risk varied depending on the prevalence of HIV/AIDS within the country. Therefore, the geopolitical regions and social segments most affected by the epidemic [36] and in which the number of HIV-Infected individuals was highest tended to present the highest absolute risks and the highest numbers of cases of late entry into care, regardless of the proportion of late entry observed for those groups and regions.

The advantage of using a approach in which the point of reference is the absolute risk is that it adds a public health perspective to the knowledge gathered to date regarding late entry into HIV care. If this new knowledge is applied, public policies designed to promote the early diagnosis of HIV infection will be more effective in reducing the number of cases of late entry into care, because they will prioritize the social segments and geopolitical regions in which the population rates of late entry are highest.

Analyses of relative risk (based on risk ratios) can facilitate the identification of social segments in which there are disproportionately large concentrations of cases of late entry into HIV care, as well as promoting the understanding of the factors that increase the probability of such late entry. Using such analyses, we observed that an increase in the proportion of late entry was independently associated with social differences (gender differences, generational differences, differences in sexual practices, and differences in terms of injection drug use), as well as contextual differences (related to the level of economic development and access to HIV testing at public health care facilities).

Our results, in terms of the social differences, are in agreement with those of studies conducted in other regions of the world [16,19-23,32], which have reported that the probability of late entry into HIV care is highest among older individuals, men (especially heterosexual men), and injection drug users. These findings can be explained by the social and cultural aspects of the AIDS epidemic [13,26] and by the barriers to health care access [18,19,27]. Therefore, late entry into HIV care tends to take on added importance in individuals who are not recognized as being at risk of HIV infection or who do not perceive themselves to be at such risk, such as older individuals [20,21]; individuals in whom the rates of treatment seeking are low, such as men [16,38]; and individuals who are socially marginalized, such as injection drug users [16,19,21]. In Brazil, this inequality is evidenced, in part, by HIV testing rates. In a home-based study conducted in Brazil in 2005 [39], the social segments in which the HIV testing rate was found to be lowest among men (28%) and among individuals ≥ 45 years of age (21%), whereas that rate was above the national average (of 34%) among women (38%), especially among those in the 25–34 year age bracket (60%). This is primarily attributable to prenatal HIV testing. Among homosexual men, the HIV testing rate was found to be below 60% [39].

In terms of the contextual factors analyzed, we found that a higher HIV testing rate translated to a lower proportion of late entry. This is underscored by the fact that, during the period evaluated, a 5% increase in the rates of HIV testing [40] was paralleled by the 4.6% reduction we observed in the proportion of late entry. That same relationship has been evaluated in studies investigating the effectiveness and cost-effectiveness of HIV testing [15,41,42], which have also shown that an increase in the availability of HIV testing corresponds to a higher rate of timely diagnosis and longer life expectancy of HIV-infected individuals.

The differences observed among municipalities of different population sizes, in terms of the proportion of late entry into HIV care, might reflect differences in the ease of access to HIV diagnosis and treatment, which tends to be restricted in smaller municipalities, where there are fewer HIV testing centers, it is more difficult to guarantee anonymity, and the social stigma associated with AIDS is often greater.

The association found between late entry into HIV care and geopolitical region with a lower GDP/increasing incidence of AIDS might indicate a two-fold relationship: a lower level of economic development creates barriers to health care access, increasing the proportion of late entry, which is, in turn, a major cause of the increase in the rate of new cases of HIV infection. This possible vicious cycle is a challenge for health care policy makers, and the promotion of early diagnosis in such regions might be an inflection point for improving our control of the epidemic.

Another important aspect of the present study was the application of three different criterion for defining late entry into HIV care. This approach allowed us to depict entry into care at the various stages in the evolution of HIV infection. Some initiatives have attempted to achieve a consensus regarding late entry into HIV care, with the objectives of increasing comparability across studies and implementing strategies of epidemiological surveillance of such late entry. In one such initiative [31], “late presentation” and “presentation with advanced HIV disease” were defined in a manner similar to that of the extended and standard criteria, respectively, employed in the present study. One advantage of using multiple criteria to define late entry was that it allowed us to characterize the contingent of HIV-infected individuals who are more likely to die because of such late entry, as occurred in nearly a third of those who were in the advanced stages of HIV infection upon entry into care. In addition, using multiple sets of criteria, we were able to highlight the potential lost opportunities to prevent new infections in others and to slow the advance of the AIDS epidemic, through the timely initiation of ART [1,2], as occurred in nearly a two thirds of all HIV-infected individuals who began to receive ART later than recommended. If we further extended the criterion to include individuals with CD4^+^ T-cell counts of 350–500 cells/mm^3^, the magnitude of these losses would be even greater, because the use of ART at that stage of infection could reduce sexual HIV transmission by 90% and the incidence of AIDS-related diseases by 40% [2].

It is noteworthy that we found not substantial statistical differences between the use of each of these distinct criteria in terms of the individual and contextual factors that led to an increase in the proportion of late entry, whether analyzed on the basis of relative risk or on the basis of population rates. Therefore, it seems unnecessary to make major changes in the policies guidelines designed to promote timely access to the diagnosis and treatment of HIV infection specifically for individuals at a given clinical stage of infection.

The results we obtained by applying different sets of criteria for defining late entry in HIV care provide evidence of the need to establish precise parameters for access to ART, based on the stage of the infection, especially in countries where there are insufficient supplies of antiretroviral drugs, in order to achieve a balance between the benefits of early initiation of ART in asymptomatic individuals [2,43,44] and the logic of prioritizing the treatment of AIDS patients in whom the risk of death is elevated because of greater clinical and immunological severity [4,6,8]. Although the ability of ART to reduce the transmission of HIV is well known [1,2], it should be borne in mind that the principal objective of ART is to improve the quality of life of HIV-infected individuals. In addition, there is no consistent evidence that the HIV/AIDS epidemic can be controlled through biomedical interventions [45].

The results obtained in the present study should be interpreted within the limits of its data and analysis, which were described in a previous study [8], in which we also analyzed the potential effects of excluding individuals in whom CD4^+^ T-cell counts were not obtained within the first six months after diagnosis. One significant limitation of the present study is the use of secondary data, which should not be taken at face value. The categories of HIV transmission require cautious interpretation, because we analyzed only the patients who progressed to AIDS, who could have characteristics that are different from those of individuals in the earlier stages of the disease. The purpose of including transmission categories here was to compensate for the lack of such information in other studies, especially in those conducted in low- or middle-income countries. Nevertheless, this study allowed us a glimpse into the complexity of late entry into HIV care, considering different criteria of classification and risk assessment. The results indicate a need to strengthen policies for timely diagnosis of HIV infection and alert us to the fact that, without changes in the proportion of late entry into HIV care, the epidemic is likely to be aggravated in less developed regions and in those with limited access to health care.

## Competing interests

The authors declare that they have no competing interests.

## Authors’ contributions

AG, MME and JCRP participated in the design of the study and performed the analysis. All authors read and approved the final manuscript.

## References

[B1] Donnell D Baeten JM Kiarie J Thomas KK Stevens W Cohen CR Heterosexual HIV-1 transmission after initiation of antiretroviral therapy: a prospective cohort analysis Lancet 2010 375 9731 2092 2098 20537376 10.1016/S0140-6736(10)60705-2 PMC2922041

[B2] Cohen MS Chen YQ McCauley M Gamble T Hosseinipour MC Kumarasamy N Prevention of HIV-1 infection with early antiretroviral therapy N Engl J Med 2011 365 493 505 21767103 10.1056/NEJMoa1105243 PMC3200068

[B3] Harrison KM Song R Zhang X Life expectancy after HIV diagnosis based on national HIV surveillance data from 25 states, United States J Acquir Immune Defic Syndr 2010 53 1 124 130 19730109 10.1097/QAI.0b013e3181b563e7

[B4] Tuboi SH Schechter M McGowan CC Cesar C Krolewiecki A Cahn P Mortality during the first year of potent antiretroviral therapy in HIV-1-infected patients in 7 sites throughout Latin America and the Caribbean J Acquir Immune Defic Syndr 2009 51 5 615 623 19430306 10.1097/QAI.0b013e3181a44f0a PMC2780368

[B5] Lawn SD Little F Bekker LG Kaplan R Campbel E Orrell C Changing mortality risk associated with CD4 cell response to antiretroviral therapy in South Africa AIDS 2009 23 3 335 342 19114870 10.1097/QAD.0b013e328321823f PMC3776050

[B6] Egger M May M Chêne G Phillips AN Ledergerber B Dabis F Prognosis of HIV-1-infected patients starting highly active antiretroviral therapy: a collaborative analysis of prospective studies Lancet 2002 360 9327 119 129 [Erratum in: Lancet 2002;360(9340):1178.] 12126821 10.1016/s0140-6736(02)09411-4

[B7] Chasombat S McConnell MS Siangphoe U Yuktanont P Jirawattanapisal T Fox K National expansion of antiretroviral treatment in Thailand, 2000–2007: program scale-up and patient outcomes J Acquir Immune Defic Syndr 2009 50 5 506 512 19223784 10.1097/QAI.0b013e3181967602

[B8] Grangeiro A Escuder MM Menezes PR Alencar R Ayres De Castilho E Late entry into HIV care: estimated impact on AIDS mortality rates in Brazil, 2003–2006 PLos One 2011 6 1 14585 10.1371/journal.pone.0014585 PMC3026775 21283618

[B9] Krentz HB Auld MC Gill MJ The high cost medical care for patients who present late (CD4 < 200 cells/microL) with HIV infection HIV Med 2004 5 2 93 98 15012648 10.1111/j.1468-1293.2004.00193.x

[B10] Fleishman JA Yehia BR Moore RD Gebo KA HIV Research Network. The economic burden of late entry into medical care for patients with HIV infection Med Care 2010 48 12 1071 1079 21063228 10.1097/MLR.0b013e3181f81c4a PMC3022268

[B11] Bartlett JG Branson BM Fenton K Hauschild BC Miller V Mayer KH Opt-out testing for human immunodeficiency virus in the United States: progress and challenges JAMA 2008 300 8 945 951 18728268 10.1001/jama.300.8.945

[B12] Obermeyer CM Osborn M The utilization of testing and counseling for HIV: a review of the social and behavioral evidence Am J Public Heath 2007 97 10 1762 1774 10.2105/AJPH.2006.096263 PMC1994175 17761565

[B13] Yazdanpanah Y Lange J Gerstoft J Cairns G Earlier testing for HIV – how do we prevent late presentation? Antiviral Therapy 2010 15 suppl 1 17 24 20442457 10.3851/IMP1526

[B14] World Health Organization HIV/AIDS. Towards universal access: Scaling up priority HIV/AIDS interventions in the health sector. Progress report 2010 2010 World Health Organization, Geneva Available from: http://whqlibdoc.who.int/publications/2010/9789241500395_eng.pdf. Accessed in 2011 (Jun 11)

[B15] Paltiel AD Weinstein MC Kimmel AD Seage GR Losina E Zhang H Expanded screening for HIV in the United States–an analysis of cost-effectiveness N Engl J Med 2005 352 6 586 595 15703423 10.1056/NEJMsa042088

[B16] Nadiaye B Salleron J Vincent A Bataille P Bonnevie F Choisy P Factors associated with presentation to care with advanced HIV disease in Brussels and Northern France BMC Infectious Diseases 2011 11 11 21226905 10.1186/1471-2334-11-11 PMC3032693

[B17] Althoff KN Gange SJ Klein MB Brooks JT Hogg RS Bosch RJ Late presentation for human immunodeficiency virus care in the United States and Canada Clin Infect Dis 2010 50 11 1512 1520 20415573 10.1086/652650 PMC2862849

[B18] Girardi E Aloisi MS Arici C Pezzotti P Serraino D Balzano R Delayed presentation and late testing for HIV: demographic and behavioral risk factors in a multicenter study in Italy J Acquir Immune Defic Syndr 2004 36 4 951 959 15220702 10.1097/00126334-200408010-00009

[B19] Sullivan AK Curtis H Sabin CA Johnson MA Newly diagnosed HIV infections: review in UK and Ireland BMJ 2005 330 7503 1301 1302 15894552 10.1136/bmj.38398.590602.E0 PMC558202

[B20] Zoufaly A Heiden M Marcus U Hoffmann C Stellbrink HJ Voss L Late presentation for HIV diagnosis and care in Germany HIV Medicine 2011 10.1111/j.1468-1293.2011.00959.x 22093171

[B21] de Olalla PG Mazardo C Sambeat MA Acaña I Knobel H Humet V Epidemiological characteristics and predictors of late presentation of HIV infection in Barcelona (Spain) during 2001–2009 AIDS Research and Therapy 2011 8 22 21729332 10.1186/1742-6405-8-22 PMC3143919

[B22] Centers for Disease Control for Prevention (CDC) Late HIV testing - 34 states, 1996–2005 MMWR Morb Mortal Wkly Rep 2009 58 24 661 665 19553901

[B23] Dickson NP McAllister S Sharples K Paul C Late presentation of HIV infection among adults in New Zealand: 2005–2010 HIV Medicine 2011 10.1111/j.1468-1293.2011.00959.x 22093231

[B24] Keiser O Anastos K Schechter M Balestre E Boulle A ART-LINC Collaboration of the International Databases to Evaluate AIDS (IeDEA) Antiretroviral therapy in resource-limited settings 1996 to 2006: patient characteristics, treatment regimens and monitoring in sub-Saharan Africa, Asia and Latin America Trop Med Int Health 2008 13 7 870 879 18373510 10.1111/j.1365-3156.2008.02078.x PMC3722496

[B25] Deblonde J De Koker P Hamers FF Fontaine J Luchters S Temmerman M Barriers to HIV testing in Europe: a systematic review Eur J Public Health 2010 20 4 422 432 20123683 10.1093/eurpub/ckp231

[B26] Carrizosa CM Blumberg EJ Melbourne FH Martinez-Donate AP Garcia-Gonzales G Lozada R Determinants and Prevalence of late HIV testing in Tijuana, Mexico AIDS Patient Care and STDs 2010 24 5 10.1089 = apc.2009.0138 10.1089/apc.2009.0138 PMC3663452 20438374

[B27] Kalichman SC Simbayi LC HIV testing attitudes, AIDS stigma, and voluntary HIV counselling and testing in a black township in Cape Town, South Africa Sex Transm Infect 2003 79 6 442 447 14663117 10.1136/sti.79.6.442 PMC1744787

[B28] Burns FM Johnson AM Nazroo J Ainsworth J Anderson J Fakoya A Missed opportunities for earlier HIV diagnosis within primary and secondary healthcare settings in the UK AIDS 2008 22 1 115 122 18090399 10.1097/QAD.0b013e3282f1d4b6

[B29] Reed JB Hanson D McNaghten AD Bertolli J Teshale E Gardner L HIV testing factors associated with delayed entry into HIV medical care among HIV-infected persons from eighteen states, United States, 2000–2004 AIDS Patient Care STDs 2009 23 9 765 773 19694550 10.1089/apc.2008.0213

[B30] Burke RC Sepkowitz KA Bernstein KT Karpati AM Myers JE Tsoi BW Why don’t physicians test for HIV? A review of the US literature AIDS 2007 21 1617 1624 17630557 10.1097/QAD.0b013e32823f91ff

[B31] Antinori A Coenen T Costagiola D Dedes N Ellefson M Gatell J Late presentation of HIV infection: a consensus definition HIV Medicine 2011 12 61 64 20561080 10.1111/j.1468-1293.2010.00857.x

[B32] Thanawuth N Chongsuvivatwong V Late HIV diagnosis and delay in CD4 count measurement among HIV-infected patients in Southern Thailand AIDS Care 2008 20 1 43 50 18278614 10.1080/09540120701439303

[B33] Fernandes JRM Acurcio FA Campos LN Guimarães Início da terapia anti-retroviral em estágio avançado de imunodeficiência entre indivíduos portadores de HIV/AIDS em Belo Horizonte, Minas Gerais, Brasil [Initiation of antiretroviral therapy in HIV-infected patients with severe immunodeficiency in Belo Horizonte, Minas Gerais State, Brazil]. Cad Saúde Pública = Rep Public Health 2009 25 6 1369 1380 10.1590/s0102-311x2009000600019 19503967

[B34] Souza-Jr PR Szwarcwald CL Castilho EA Delay introducing antiretroviral therapy in patients infected by HIV in Brazil, 2003–2006 Clinics (São Paulo) 2007 62 5 579 584 17952318

[B35] Lucena FFA Fonseca MGP Sousa AIA Coeli CM O relacionamento de bancos de dados na implementação da vigilância da AIDS. Relacionamento de dados e vigilância da AIDS [Applying record linkage in AIDS surveillance] Cad Saúde Colet (Rio J.) 2006 14 2 305 312

[B36] Grangeiro A Escuder MM Castilho EA Magnitude and trend of the AIDS epidemic in Brazilian cities, from 2002 to 2006 Rev Saude Publica 2010 44 3 430 440 20464259 10.1590/s0034-89102010005000013

[B37] Ministério da Saúde Secretaria de Vigilância em Saúde Departamento Nacional de DST Aids e Hepatites Virais. Boletim Epidemiológico Aids-DST 2011, Brasília Ano VIII, No. 1. Available from: http://www.aids.gov.br/publicacao/2011/boletim_epidemiologico_2011. Accessed in 2011 (Dez 20)

[B38] Nhac-Vu H-T Giard M Phong N-D Vanhems P Risk factors for delayed HIV diagnosis at the hospital of tropical diseases in Ho Chi Minh City, Vietnam International Journal of STD & AIDS 2010 21 802 805 21297086 10.1258/ijsa.2010.010045

[B39] França Junior I Calazans G Zucchi EM Grupo de Estudos em População, Sexualidade e Aids. Mudanças no âmbito da testagem anti-HIV no Brasil entre 1998 e 2005 [Change in HIV testing in Brazil between 1998 and 2005] Rev Saude Publica 2008 42 1 84 97 18660928 10.1590/s0034-89102008000800011

[B40] Brazilian Ministry of Health Health Surveillance Secretariat National Programme STD and AIDS Targets and Commitments made by the Member-States at the United Nation General Assembly Special Session on HIV/AIDS. UNGASS – HIV/AIDS. Brazilian Response: 2005/2007. Country Progress Report 2008 National Programme STD and AIDS, Brasília Available from: http://data.unaids.org/pub/report/2008/brazil_2008_country_progress_report_en.pdf Accessed in 2011 (May 11)

[B41] Sanders GD Bayoumi AM Sundaram V Bilir SP Neukermans CP Rydzak CE Cost-effectiveness of screening for HIV in the era of highly active antiretroviral therapy N Engl J Med 2005 352 570 585 15703422 10.1056/NEJMsa042657

[B42] Tole SP Sanders GD Bayoumi AM Galvin CM Vinichenko TN Brandeau ML Cost-effectiveness of voluntary HIV screening in Russia Int J STD AIDS 2009 20 1 46 51 19103893 10.1258/ijsa.2008.008128 PMC2981087

[B43] Severe P Juste MA Ambroise A Eliacin L Marchand C Apollon S Early versus standard antiretroviral therapy for HIV-infected adults in Haiti N Engl J Med 2010 363 3 257 265 20647201 10.1056/NEJMoa0910370 PMC3676927

[B44] Siegfried N Uthman OA Rutherford GW Optimal time for initiation of antiretroviral therapy in asymptomatic, HIV-infected, treatment-naive adults Cochrane Database Syst Rev 2010 3 CD008272 20238364 10.1002/14651858.CD008272.pub2 PMC6599830

[B45] Dodd PJ Garnett GP Hallett TB Examining the promise of HIV elimination by ‘test and treat’ in hyper-endemic settings AIDS 2010 24 5 729 735 20154580 10.1097/QAD.0b013e32833433fe PMC2852517

